# Pannexin 1 channels in skeletal muscles

**DOI:** 10.3389/fphys.2014.00139

**Published:** 2014-04-11

**Authors:** Luis A. Cea, Manuel A. Riquelme, Anibal A. Vargas, Carolina Urrutia, Juan C. Sáez

**Affiliations:** ^1^Departamento de Fisiología, Pontificia Universidad Católica de ChileSantiago, Chile; ^2^Centro Interdisciplinario de Neurociencias de Valparaíso, Universidad de ValparaísoValparaíso, Chile; ^3^Department of Biochemistry, University of Texas Health Science CenterSan Antonio, TX, USA

**Keywords:** potentiation, sarcolemma leakage, muscular plasticity, phosphorylation, ATP

## Abstract

Normal myotubes and adult innervated skeletal myofibers express the glycoprotein pannexin1 (Panx1). Six of them form a “gap junction hemichannel-like” structure that connects the cytoplasm with the extracellular space; here they will be called Panx1 channels. These are poorly selective channels permeable to ions, small metabolic substrate, and signaling molecules. So far little is known about the role of Panx1 channels in muscles but skeletal muscles of Panx1^−/−^ mice do not show an evident phenotype. Innervated adult fast and slow skeletal myofibers show Panx1 reactivity in close proximity to dihydropyridine receptors in the sarcolemma of T-tubules. These Panx1 channels are activated by electrical stimulation and extracellular ATP. Panx1 channels play a relevant role in potentiation of muscle contraction because they allow release of ATP and uptake of glucose, two molecules required for this response. In support of this notion, the absence of Panx1 abrogates the potentiation of muscle contraction elicited by repetitive electrical stimulation, which is reversed by exogenously applied ATP. Phosphorylation of Panx1 Thr and Ser residues might be involved in Panx1 channel activation since it is enhanced during potentiation of muscle contraction. Under denervation, Panx1 levels are upregulated and this partially explains the reduction in electrochemical gradient, however its absence does not prevent denervation-induced atrophy but prevents the higher oxidative state. Panx1 also forms functional channels at the cell surface of myotubes and their functional state has been associated with intracellular Ca^2+^ signals and regulation of myotube plasticity evoked by electrical stimulation. We proposed that Panx1 channels participate as ATP channels and help to keep a normal oxidative state in skeletal muscles.

## Pannexin 1 based channels: structure and expression

Myotubes (Buvinic et al., 2009) and innervated myofibers (Cea et al., 2013; Riquelme et al., 2013) express pannexin1 (Panx1). Panx genes (PANX) are a small family; three of them have been cloned from mammals and are called PANX1, PANX2, and PANX3. They are orthologs of invertebrate innexins (Inxs) (Ambrosi et al., 2010), which form gap junction channels allowing the direct cytoplasm communication between adjacent cells (Sáez et al., 2003). Presently, it has been proposed that gap junction channels of chordates have to be formed exclusively by connexins (Cxs) (Dahl and Keane, 2012). Gap junction channel formation requires plasma membrane proximity of two cells and the docking of two hemichannels (half of a gap junction channel), each one provided by one of two adjacent cells (Sáez et al., 2003). However, mammalian Panxs and Inxs do not share significant gene and protein sequence homology with chordate Cxs. Nevertheless, Panx channels share some pharmacological properties with Cx-based channels and present some differences in sensitivity to different compounds and conditions including Probenecid, carbenoxolone, flufenamic acid, La^3+^ and cytoplasmic acidification (D'Hondt et al., 2009). Panx1 channels are also inhibited by brilliant blue G, a P2X receptor blocker, and by the food dye FD&C blue N° 1 [Brilliant Blue FCF (BB FCF) or Blue 1] (Wang et al., 2013), present in Blue Gatorade (see in nutritional facts on official web site www.gatorade.com) and Blue M&M (see in ingredients on official web site www.mms.com). Also, they share some permeability properties with Cx hemichannels, since they are permeable to small positively [i.e., ethidium (Etd^+^), DAPI] and negatively (i.e., gluconate, glutamate, ATP, and aspartate) charged organic molecules and inorganic ions (Na^+^, K^+^, Ca^2+^, and Cl^−^). In addition, Panx1 forms gap junction channels in exogenous expression systems including Xenopus *laevis* oocytes and some mammalian cells lines (Vanden Abeele et al., 2006). Nevertheless, up to the present there is no *in vivo* evidence that Panxs form gap junction channels, as compared to the evidence for Cxs or Inxs (Dahl and Keane, 2012). Therefore, these channels seem to work exclusively as channels in the nonjunctional membrane, allowing the exchange of molecules between the cytoplasm and the extracellular space. Panx1 channels present in the reticular system and the plasma membrane are activated by positive membrane potentials, high extracellular K^+^, extracellular ATP via P2 receptors, stretch-induced membrane deformation and posttranslational modifications (D'Hondt et al., 2013).

Panx1 is ubiquitously expressed in several tissues. Different levels of mRNA are detected in northern blots, being strongly expressed in heart, skeletal muscle, testis, and ovary. Medium levels are present in brain (Bruzzone et al., 2003), placenta, thymus, kidney, prostate, and small intestine, and low to almost undetectable levels are found in lung, liver, pancreas, spleen, colon, and peripheral blood (Baranova et al., 2004). The pattern of expression of the other two Panxs is completely different; Panx2 is preferentially expressed in brain (Baranova et al., 2004) and Panx3, mostly expressed in bone and skin (Sandilos and Bayliss, 2012). Panx1 channels are oligomeric structures formed by 6 subunits of Panx1 protein (Boassa et al., 2007). Panx1 shows similar membrane topology than Cxs and is characterized by four transmembrane (TM) segments, two extracellular loops, and cytoplasmic localization of both amino and C-termini. Panxs 1 and 3 are glycoproteins, unlike Cxs that are not glycosylated. It has been proposed that glycosylation of Panxs prevents formation of gap junction channels (Boassa et al., 2008). Panx1 and Panx3 suffer N-linked glycosylation at Asp254 present in the second extracellular loop and Asp71 found at the first extracellular loop, respectively. However, a fraction of Panx1 mutant that is not glycosylated still traffics to the cell surface, suggesting that Panx1 glycosylated protein could interact and form channels with unglycosylated forms and/or that the unglycosylated Panx1 fraction forms gap junction channel-like structures (Penuela et al., 2009).

Structural analyses of the lining residues of the Panx1 pore using the substituted cysteine accessibility method (SCAM) have revealed that the TM1 region and domains of the first extracellular loop (E1) are exposed to the channel lumen. Also, C-terminal amino acids substitution and reagent perturbation suggest the contribution of this segment to the permeation pathway (Wang and Dahl, 2010). Panx1 channels activated by positive membrane potentials show several substates. The full open state presents a characteristically high conductance in different cell types including Xenopus oocytes, insulinoma cells and cardio myocytes (Bao et al., 2004; Iglesias et al., 2009; Kienitz et al., 2011). However, recent studies using different Panx1 transfectants revealed a characteristic unitary conductance of ~60 pS (Ma et al., 2009; Romanov et al., 2012). Moreover, the permeability of Panx1 channels has been recently shown to be negligible to anions exceeding 250 Da, which would exclude ATP (Romanov et al., 2012). This apparent controversy might be explained by Panx1 channel variations due to pore properties, such as diameter and length changes caused by different interactions with other cellular proteins or due to different post-translational modifications in different cell types. Thus further studies maybe required to clarify this issue.

## Activation of Panx1 channels

Several stimuli increase the activity of Panx1 channels. Among them are: increase in extracellular K^+^ concentration, positive membrane voltage over +40 mv, extracellular ligands, such as ATP [which activates Panx1 channels in micromolar concentrations (Locovei et al., 2006) but inhibit them in milimolar concentrations (Qiu and Dahl, 2009)], that enhance the intracellular free Ca^2+^ levels including like P2Y_1−2_ receptors coupled to Gq proteins or P2X_7_ receptors that are non-selective cationic channels with a slow kinetic of inactivation (Locovei et al., 2006, 2007; Iglesias et al., 2008). Panx1 channel opening has been induced by glutamate through NMDA receptor activation in neurons (Thompson et al., 2008; Orellana et al., 2011), neuronal stress induced by oxygen glucose deprivation (Thompson et al., 2008), hypertonic stress in lymphocytes (Woehrle et al., 2010) and increase in intracellular free Ca^2+^ levels induced by Ca^2+^ ionophore (Locovei et al., 2006). Although Panx1 does not have putative Ca^2+^ biding sites, it possesses multiple phosphorylation consensus sites in the C-terminal tail to several serine and threonine kinases (Penuela et al., 2007; Riquelme et al., 2013). A possible mechanism of activation of Panx1 channels that involves phosphorylation has been recently suggested. During repetitive skeletal muscle contraction, the Panx1 channel activity increases and the state of phosphorylation of Panx1 Ser and Thr residues is also increased (Riquelme et al., 2013). In contrast, phosphorylation of Tyr residues in Panx1 has not been detected yet (Iglesias et al., 2008; Riquelme et al., 2013). A protein phosphorylation-dependent activation could be followed by inactivation via a phosphoprotein phosphatase or by phosphorylation of a different amino acid residue by another protein kinase with less Ca^2+^ affinity, followed by complete dephosphorylation via a protein phosphatase.

An alternative mechanism of Panx1 channel activation in skeletal muscle could be a direct protein-protein dependent mechanism (Panx1 channel/dihydropyridine receptor) mediated by conformational changes of voltage activated dihydropyridine receptors induced by depolarizing membrane potentials. In support of this view it is possible to say that electrical stimulation induces ATP release and uptake of Etd^+^ and fluorescent glucose derivatives in myofibers, indicating opening of Panx1 channels (Riquelme et al., 2013). Also, myotubes lacking the Cav1.1-α1 subunit released almost no ATP upon electrical stimulation, suggesting that Cav1.1 plays a critical role in this process (Jorquera et al., 2013).

## Role of Panx1 based channels in normal skeletal muscles

### Possible role of Panx1 channels in muscular ontogeny

Skeletal muscles develop through a process partially coordinated by extracellular signaling. The coordinate response of cell groups includes the myogenic commitment of mesodermal pluripotent cells, myoblast alignment and fusion. In mice, this process requires the presence of Cx43 expression and functional gap junction channels (Kalderon et al., 1977; Proulx et al., 1997; Araya et al., 2003). In rats, these channels disappear at about 1 week postnatal age when skeletal muscles become innervated (Cea et al., 2012).

The acquisition of myogenic commitment requires increase of [Ca^2+^]_i_, and activation of calcineurin, a Ca^2+^-dependent protein phosphatase that induces expression of the transcription factor myf-5 (Friday and Pavlath, 2001). Increases in [Ca^2+^]_i_ could be induced by activation of P2 receptors with extracellular ATP/ADP. In addition, activation of P2X receptors 2, 4 or 7 increases the cell membrane permeability to small molecules, including Lucifer yellow, Etd^+^ and YO-PRO-1, in diverse cell types such as myoblasts and macrophages (North, 2002; Araya et al., 2005; Pelegrin and Surprenant, 2006). However, Panx1 has been proposed to mediate the plasma membrane permeabilization to dyes after activation of P2X/Y receptors (Pelegrin and Surprenant, 2006; Locovei et al., 2006, 2007).

Treatment with a concentration of β-glycyrrhetinic acid that blocks connnexin-based channels (gap junction channels and hemichannels) and Panx1 channels (Bruzzone et al., 2005) prevents the expression of myogenin and MRF4, two transcription factors that promote myogenesis and myotubes formation (Proulx et al., 1997). However, treatment with octanol, blocker of Cx-based channels but not Panx1 channels (Bruzzone et al., 2005; Pelegrin and Surprenant, 2006), does not block myogenesis as evaluated by the expression of the pro-myogenic transcription factor Myf-5 (Proulx et al., 1997). Thus, the presence of functional Panx1 channels might be enough to promote commitment and myogenesis *in vitro*, and probably Cx hemichannels and/or other Ca^2+^ channels have a redundant role that overcomes the lack of Panx1, since Panx1^−/−^ mice do not show evident muscular phenotype changes. In agreement with the role of Cx-based channels coordinating the commitment of myoblast, Cx43 deficient muscles show a delay regeneration response after BaCl_2_-induced damage (Araya et al., 2005).

## Potentiation of muscular contraction

**Figure 1:**
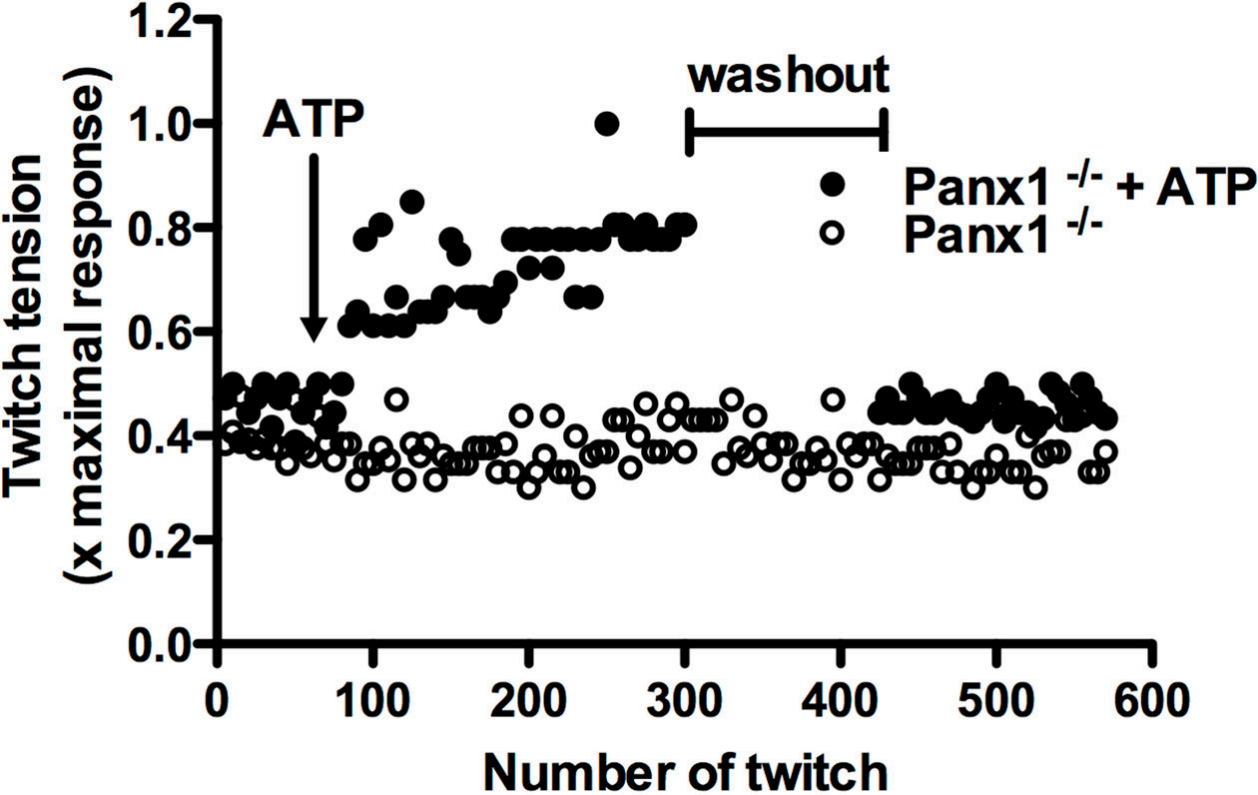
**Exogenous ATP reverses the absence of potentiation of muscular contraction in Panx1 deficient mice**. Potentiation of muscular contraction was induced by electrical transmural stimulation (0.03 Hz, 45 V, 100 ms stimuli duration) in isolated Soleus muscle from Pannexin 1 deficient mice (Panx1^−/−^), and exogenous ATP was applied (200 μ M, final concentration, closed circles) after 65 twitches. Then, the muscle was rinsed during 2 min and the potentiation response was re-evaluated. In addition, the potentiation response with the same number of twitches was evaluated in absence of ATP and washout (open circles).

The force generated in muscular contraction increases after repetitive twitches. This response is called potentiation and depends on an increase of intracellular free Ca^2+^ concentration (Sandonà et al., 2005; Zhi et al., 2005) due to Ca^2+^ release from intracellular stores and Ca^2+^ inflow from the extracellular space in fast and slow twitch muscles, respectively. In the latter, Ca^2+^ uptake depends on the activation of purinergic ionotropic P2X_4_ receptors (Sandonà et al., 2005), which are highly expressed in this type of muscle. For fast twitch muscles, the potentiation response is independent of the extracellular Ca^2+^ concentration (Louboutin et al., 1996), but requires extracellular ATP. Accordingly, extracellular ATPases inhibit the potentiation response (Sandonà et al., 2005) and, together with the absence of P2X receptors, have led to suggest that the potentiation response in these muscles is mediated by activation of metabotropic P2 receptors as P2Y_1_ receptors (Riquelme et al., 2013). Recently, Panx1 channels were proposed as a possible pathway for ATP release in skeletal muscles (Riquelme et al., 2013). In normal adult skeletal muscle, Panx1 was localized in the sarcolemma of the T-tubules system (Cea et al., 2012; Riquelme et al., 2013; Jorquera et al., 2013). Moreover, Panx1 channels were activated by electrical stimulation allowing the uptake of glucose (a fluorescent glucose analog, 2-NBDG) and release of ATP, which are necessary for potentiation of muscular contraction (Sandonà et al., 2005; Riquelme et al., 2013). In addition, the absence of Panx1 in myofibers of Panx1 deficient mice (Panx1^−/−^ mice) does not exhibit potentiation of muscle contraction induced by electrical stimulation. Therefore, it was suggested that this response is due to the absence of ATP release (Riquelme et al., 2013). In agreement with this notion, the lost potentiation response of skeletal muscles from Panx1^−/−^ mice can be reversed by addition of exogenous ATP to the bath, shown for the first time here (Figure 1). This finding confirms that Panx1 channels are necessary for the potentiation response, and is consistent with the hypothesis that potentiation *in vivo* could be due to permeability of Panx1 channels to ATP, at least in activated skeletal muscles. It further suggests that the presumptive Panx1 channel-dependent mechanism for ATP release is not compensated by other pathways of ATP release, or other possible mechanisms of potentiation.

## Role of Panx1 channels in pathological conditions of skeletal muscles

**Figure 2:**
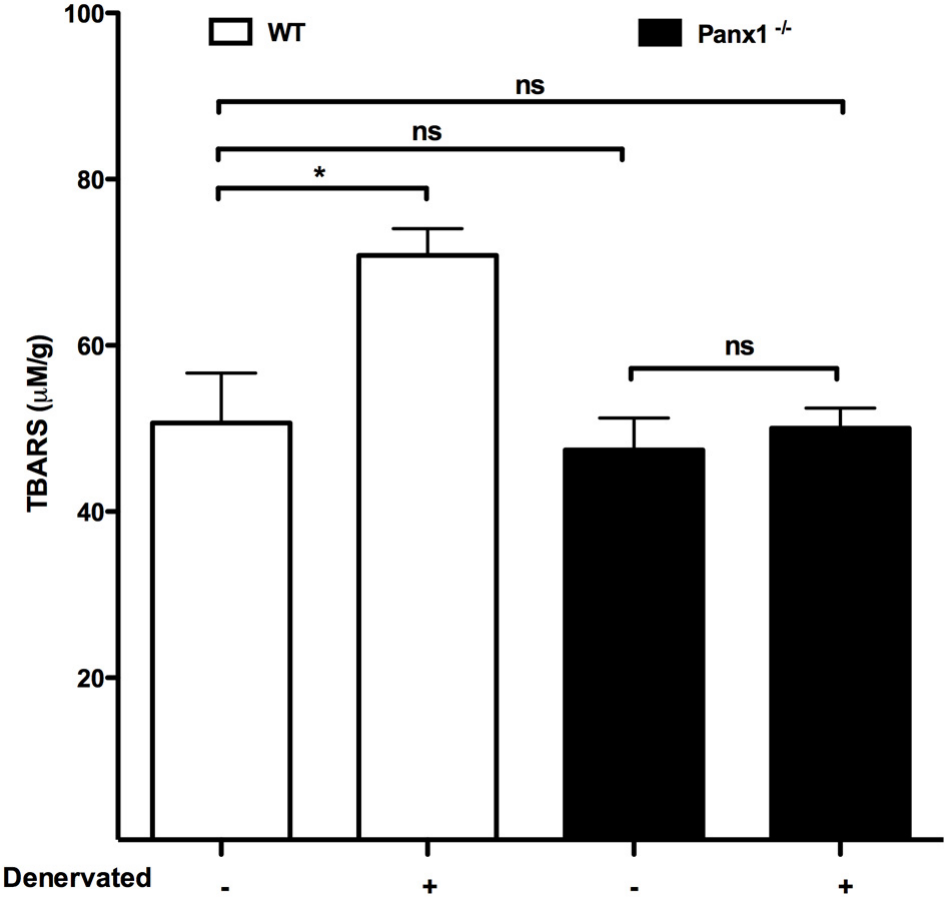
**The absence of Panx1 prevents the increase in levels of thiobarbituric reactive substances in denervated skeletal muscles**. Seven days after denervation gastrocnemius muscles were isolated from wild type (wt, white bar), or Panx1 deficient mice (Panx1^−/−^, black bar), and levels of thiobarbituric acid reactive substances (TBARS) were evaluated in denervated vs. not denervated muscles. ^*^P < 0.05; ns, not significant.

Recently, denervation was shown to induce *de novo* expression of Cx-based hemichannels that mediate a drastic increase in sarcolemma permeability and leads to muscular atrophy (Cea et al., 2013). In addition, it was found that Panx1 channels do not play a crucial role on this phenomenon, since muscles of Panx1^−/−^ mice showed similar increase in membrane permeability and atrophy to those observed in denervated muscles of wild type animals (Cea et al., 2013). Until now, this is the only work published in which a possible pathological role of Panx1-based channels in skeletal muscles has been evaluated. In addition, it was analyzed the production of thiobarbituric reactive substances (TBARS), including malondialdehyde (MDA), as a measure of oxidative stress in denervated skeletal muscles of Panx1^−/−^ mice because denervation is known to increase the skeletal muscle levels of reactive oxygen species (ROS) (Abruzzo et al., 2010). We found, in this work, that the absence of Panx1 does not affect the basal levels of TBARS but prevented the increase in TBARS levels present in 7-day denervated wild type muscles (Figure 2), suggesting that Panx1 channels might allow loss of reducing agents and thus their absence would protect against ROS generation. Alternatively, constitutive Panx1^−/−^ muscles might have developed more antioxidant phenotype and thus could be more resistant to the denervation-induced ROS generation.

## Skeletal muscle plasticity

The skeletal muscle activity induces remodeling of structure and functional performance of myofibers, changing the muscular force output, endurance and contractile velocity with respect to a functional demand (Tavi and Westerblad, 2011). All these changes are called skeletal muscle plasticity. This adaptive process depends on the frequency of repetitive fiber contraction, activation of intracellular signal pathways and gene expression that promotes the establishment of new myofiber characteristics (Tavi and Westerblad, 2011). The physiological importance of skeletal muscle plasticity is evident; however, the molecular mechanisms involved in muscle plasticity remain elusive. Electrical stimuli at frequencies that activate Panx1 channels (20 Hz) induce the expression of a molecular marker that reflects the transition between a fast to slow myofiber (Jorquera et al., 2013). Panx1 channels present in the T-tubule membrane are regulated for the dihydropyridine receptors; with high frequencies (90 Hz) the activity of Panx1 channels is low and the expression of plasticity marker in myofibers does not change (Jorquera et al., 2013). The electrical stimulation induces ATP release through Panx1 channels, eliciting an IP_3_-dependent intracellular Ca^2+^ signal (Eltit et al., 2006) which is directly associated with gene expression changes (Semsarian et al., 1999; Jaimovich and Carrasco, 2002; Carrasco et al., 2003; Buvinic et al., 2009). These responses depend on the activation of P2 receptors by extracellular ATP because apyrase, an ATP hydrolase, or suramin, a general P2 receptors inhibitor, blocks both signals. The metabotropic Ca^2+^ signal induced by extracellular ATP was prevented by Panx1 channel inhibitors (^10^Panx1 and oleamide) that reduced the calcium transients and the ATP release (Buvinic et al., 2009). Consequently, it was proposed that a train of action potentials with a defined frequency induces Ca^2+^ release events that differentially activate Ca^2+^-dependent signaling pathways, which determine the expression of genes responsible for the slow or fast muscle phenotype (Tavi and Westerblad, 2011). These signaling pathways include calcineurin–NFAT-, Ca^2+^/calmodulin-dependent kinases II and IV- and protein kinase C-dependent pathways (Tavi and Westerblad, 2011). These Ca^2+^ signals lead to muscular plasticity by modulating the expression of several genes including IL-6 and c-*fos*, and the switch between troponin isoform from fast to slow fiber (Buvinic et al., 2009; Jorquera et al., 2013).

## Concluding remarks

Panx1 channels are involved in several relevant physiological skeletal muscle processes, such as potentiation of skeletal muscle contraction since they do not occur in the absence of these channels. Currently, Panx1 channels have been clearly involved in potentiation of contraction of skeletal muscles. In addition, Panx1 channels appear to mediate the release of ATP involved in muscle remodeling. People who are active in sports should be cautious with food and drink additives that could reduce the Panx1 channel activity and consequently the potentiation response of muscle contraction. Moreover, to further understand the possible role of Panx1 channels in pathologic responses it may be needed to develop highly selective Panx1 channels inhibitors and/or acute down regulation of Panx1 expression using inducible KO animals or transfections with siRNA or morpholines. Finally, Panx1 channels act as ATP channels and the absence prevent the induction of reactive oxygen species; measured as levels of TBARS in this work (Figure 2), suggesting that Panx1 channels are relevant to keep healthy skeletal muscles.

### Conflict of interest statement

The authors declare that the research was conducted in the absence of any commercial or financial relationships that could be construed as a potential conflict of interest.
